# Degradation of AIMP1/p43 Induced by Hepatitis C Virus E2 Leads to Upregulation of TGF-β Signaling and Increase in Surface Expression of gp96

**DOI:** 10.1371/journal.pone.0096302

**Published:** 2014-05-09

**Authors:** Min Soo Kim, Sunghoon Kim, Heejoon Myung

**Affiliations:** 1 Department of Bioscience and Biotechnology, Hankuk University of Foreign Studies, Yong-In, Gyung-Gi Do, Korea; 2 Medicinal Bioconvergence Research Center, College of Pharmacy, Seoul National University, Seoul, Korea; Utah State University, United States of America

## Abstract

Hepatitis C virus (HCV) causes chronic hepatitis leading to liver fibrosis and autoimmune diseases. AIMP1/p43 is a multifunctional protein initially known as a cofactor of aminoacyl tRNA synthetase complex. Its function includes negative regulation of TGF-β signaling and suppression of Lupus-like autoimmune disease by inhibition of surface expression of gp96. HCV E2 was shown to directly interact with AIMP1/p43 by GST pulldown assay and coimmunoprecipitation. Their subcellular colocalization was observed in an immunofluorescence confocal microscopy. We showed that HCV E2 led to degradation of AIMP1/p43 in two ways. First, in the presence of HCV E2, endogenous AIMP1/p43 was shown to be degraded in an ubiquitin-dependent proteasome pathway. Second, grp78, an ER chaperone, was shown to interact with and stabilize AIMP1/p43. And HCV E2 inhibited this interaction leading to reduction of cellular AIMP1/p43. The degradation of AIMP1/p43 by HCV E2 resulted in increase of TGF-β signaling and cell surface expression of gp96. Thus we suggest that these are novel mechanisms responsible for liver fibrosis and autoimmune diseases caused by HCV.

## Introduction

Hepatitis C virus (HCV) causes chronic hepatitis in human [10]. The virus often escapes from host immune system and more than 70% of infected patient maintains prolonged infection states [35]. It leads to liver cirrhosis and hepatocellular carcinoma [32], [36]. The virus is also reported to be involved in immune-pathological states including autoantibody production, autoimmune thyroid disorder, mixed cryoglobulinemia, and B cell lymphoma [9].

E2 is an HCV envelope protein which is important for viral entry. CD81, SR-B1, claudin-1, and occludin are known host cell surface receptors and mediate viral endocytosis [4], [8], [30], [33]. The fusion of viral and cellular membranes at low pH discharges viral genome into cytosol. The genome is a 9.6 kilobase positive single strand RNA and is translated into a polyprotein by host translation machinery in a cap-independent fashion [26]. The polyprotein is later cleaved by host and viral proteases into functional proteins [2], [38]. E2 is also involved in regulation of cellular signaling. It interacts with cellular RNA-activated protein kinase (PKR) and inhibits the phosphorylation of translation initiation factor 2 subunit α (eIF2α). This leads to inhibition of antiviral effect of interferon mediated by eIF2α [29]. It is also reported that E2 leads to the overexpression of two ER chaperones, gp96 and grp78 [20]. gp96 is another name for grp94. Overexpression of gp96 results in inhibition of apoptosis, thus maintaining prolonged infection states [17].

AIMP1/p43 is one of the cofactors of aminoacyl tRNA synthetase complex and has both proinflammatory and antiangiogenic functions [15], [18], [27], [28]. It binds to and stabilizes Smad ubiquitination regulatory factor 2 (Smurf2) [19]. Smurf2 is an E3 ligase of TGF-β receptor II [25]. The ubiquitination and proteasomal degradation of the receptor inhibits TGF-β signaling. The degradation of Smurf2 by Smad7 leads to loss of inhibition of TGF-β signaling. AIMP1/p43 and Smad7 compete each other for binding to Smurf2 and balance the level of TGF-β signaling. AIMP1/p43 also interacts with gp96 and blocks translocation of gp96 to cell surface [12]. AIMP1/p43-depleted cell shows increased cell surface expression of gp96. Cell surface gp96 activates dendritic cells and leads to autoimmune disease [22]. AIMP1/p43 knockout mice show lupus-like autoimmune disease phenotype [12].

Based on our initial observation of interaction between HCV E2 and cellular AIMP1/p43, we present novel mechanisms how HCV causes liver fibrosis and autoimmune disease in this report.

## Materials and Methods

### Plasmids

pUC-JFH1 containing full length 2a type HCV genome is a kind gift from T. Wakita. E2 (amino acids 384-750 of JFH1) was cloned into pcDNA3-HA vector (Invitrogen) with N-terminal HA tag or pCMV-tag 2B vector (Stratagene) with N-terminal FLAG tag. HCV E2, core-E1 and core-E1-E2 were separately cloned into pcDNA4 V5-HisA vector (Invitrogen) with C-terminal V5 tag. Soluble fraction of 1b type (H77) E2 (amino acids 384-661) was pEF6A-V5-6XHis vector for proximity ligation assay.

### Antibodies and reagents

AIMP1/p43 antibodies are describes elsewhere [17]. HA, FLAG, and β-tubulin antibodies were purchased from Sigma. grp78 and gp96 antibodies were purchased from Santa Cruz Biotechnology. GST antibody was from Amersham Bioscience and V5 antibody was from Invitrogen. HRP-conjugated mouse or rabbit secondary antibody and FITC- or TRITC-conjugated secondary antibody were purchased from Jackson ImmunoResearch Laboratories. TGF-β was from R&D Systems and MG132 was from Calbiochem.

### Cell culture and transfection

Human hepatocyte Huh-7 cell line was used throughout the study. HEK 293 cell line was used for coimmunoprecipitation. Both cells were grown in Dulbecco's modified eagle's medium (Hyclone) with 10% fetal bovine serum, 100 µg/ml penicillin G, and 100 µg/ml streptomycin (Hyclone) in CO_2_ incubator at 37°C. Bjab cells were grown in RPMI1640 medium (Hyclone) with 10% fetal bovine serum, 100 µg/ml penicillin G, and 100 µg/ml streptomycin (Hyclone). Cells were seeded into 6 well plate at 1×10^5^ cells/well and plasmids were transfected with Lipofectamin 2000 (Invitrogen) in 24 hours. Media were changed 6 hours after transfection.

### Western blot

Samples were loaded on a 12% polyacrylamide gel and electrophoresis was carried out. Bands were transferred to nitrocellulose membrane (Millipore). The membrane was incubated in blocking buffer (3% skim milk in TBST) at room temperature for 1 hour with rocking. The membrane was washed with TBST for three times and incubated with primary antibody at room temperature for 90 minutes. After wash with TBST for three times, HRP conjugated secondary antibody was added and the mixture was incubated for 1 hour. The membrane was washed with TBST three times and the bands were detected with ECL detection kit (Amersham).

### GST pulldown assay

Cells were lysed with RIPA buffer at 4°C for 30 minutes. Purified GST or GST-E2 was added to the lysate and incubated at 4°C for 2 hours. 40 µl of glutathione sepharose 4B beads (Amersham) was added and the total volume was adjusted to 200 µl with HNGT buffer (20 mM HEPES, pH 7.5, 150 mM NaCl, 10% glycerol, 0.1% Triton X-100) and the mixture was incubated at 4°C overnight. The mixture was washed with 1 ml of HNGT buffer five times and boiled in SDS sample buffer for 5 minutes before SDS-PAGE and western blot analysis.

### Coimmunoprecipitation

Plasmids were transfected to HEK 293 cells and RIPA buffer was used for cell lysis in 36 hours. 2 µg of antibody for immunoprecipitation was added and the mixture was incubated at 4°C for 2 hours with rocking. IgG antibody was used as a control for nonspecific precipitation. 25 mg of protein G was added and the mixture was washed with lysis buffer five times before loading on a SDS-PAGE.

### In situ proximity ligation assay (PLA)

24 hours after transfection of plasmid expressing V5-tagged HCV E2 and plasmid expressing AIMP1/p43 into HEK293 cells, cells were fixed with 3.7% formaldehyde for 15 minutes. Then cells were treated with permeabilization solution (0.5% Triton X-100 in phosphate buffered saline) for 5 minutes. Then cells were washed with washing solution (0.1% Triton X-100 in phosphate buffered saline) for 10 minutes three times. Proximity ligation assay was done as instructed in Duolink in situ kit (Sigma, USA). Primary antibodies used were anti-V5 for detection of E2 and anti-AIMP1/p43 for detection of AIMP1/p43. Laser scanning fluorescence confocal microscope (Zeiss, Germany) was used for detection of fluorescence image.

### Immunostaining

Cells were fixed in fixing solution (3.7% paraformaldehyde and 1 N NaOH in PBS) at room temperature for 15 minutes. Afterwards the cells were treated with permeabilization solution (0.5% Triton X-100 in PBS) for 5 minutes. The mixture was washed with washing solution (0.1% Triton X-100 in PBS) for three times and then treated with blocking solution (10% calf serum and 0.5% gelatin in PBS) for 30 minutes. The mixture was washed with washing solution three times and AIMP1/p43 antibody was added and incubated at room temperature for 1 hour. After washing three times, secondary antibody was added and the mixture was incubated for at room temperature for 1 hour. The mixture was mounted with mounting solution (10 mg/ml p-phenylenediamine and 20% glycerol, pH 8.5) after three times' wash and observed in laser scanning confocal microscope.

### siRNA

Duplex siRNAs for knockdown of grp78 were constructed (Bioneer, Korea). The sequence for the negative control was 5′- CCU ACG CCA CCA AUU UCG U dTdT-3′. The sequence for grp78 knockdown was 5′-CAC AGA UUG AAG UCA CCU U dTdT-3′. 100 nM siRNA was transfected to Huh-7 cells using Lipofectamin 2000 (Invitrogen) in a 6 well plate. Cells were harvested and subjected to analysis in 72 hours.

### Fluorescent activated cell sorting (FACS)

Cells were washed with 1X PBS buffer and resuspended in FACS buffer (2% FBS, 1% GSA, and 0.1% sodium azide in PBS). 1∶20 diluted anti-gp96 antibody was added and the mixture was incubated at room temperature for 30 minutes. After three times' wash with washing buffer (0.5% BSA in PBS), 1∶50 diluted anti-rabbit FITC antibody was added and the mixture was incubated at roon temperature for 30 minutes. FACS (BD Bioscience) analysis was done after three times' wash with washing buffer followed by resuspension in PBS solution.

### Luciferase assay

p3TP-lux [41] was used as a reporter. pCH110 harboring β-galactosidase gene was used as a transfection normalization plasmid. 6 hours after transfection, TGF-β was added at a concentration of 4 ng/ml and cells were incubated for 20 hours. After washing with 1X PBS, cells were lysed with passive lysis buffer (Promega) and luciferase activity was measured using Luciferase reporter assay system (Promega).

## Results

### HCV E2 interacts with cellular AIMP1/p43

**Figure 1:**
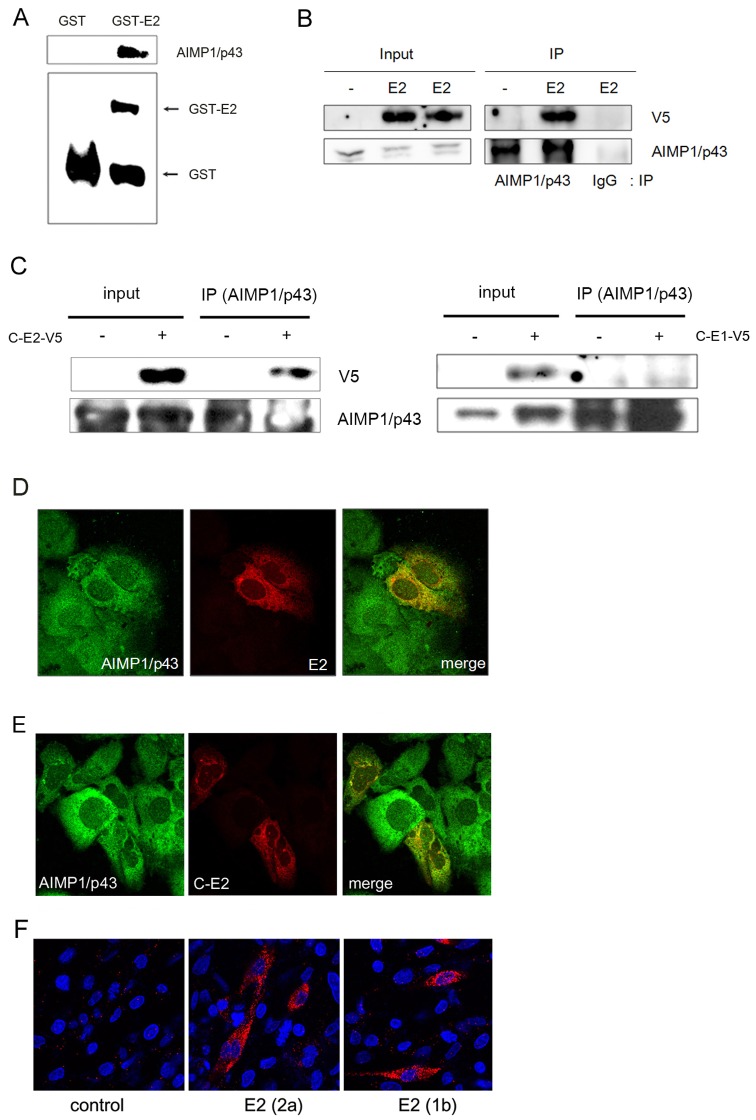
HCV E2 and cellular AIMP1/p43 interacted with each other. A. GST pulldown assay showed interaction of the two proteins. Purified GST-E2 was used for binding to expressed AIMP1/p43 in HEK 293 cells. Purified GST was used as a control. B. V5-tagged E2 was expressed in HEK 293 cells and immunoprecipitation was done with anti-V5 antibody. IgG was used as a control. C. C-terminal V5-tagged core-E1 or core-E1-E2 were expressed in HEK 293 cells and immunoprecipitation was done with anti-AIMP1/p43 antibody. D. The two proteins showed subcellular colocalization. Plasmid expressing AIMP1/p43 and plasmid expressing V5-E2 were transfected to Huh-7 cells. 24 hours' after transfection, cells were treated with 10 µM MG132 for 6 hours. Cells were immunostained with anti-AIMP1/p43 antibody (green) and anti-V5 antibody (red) and observed under confocal microscope. E. Native E2 and AIMP1/p43 showed subcellular colocalization. Plasmid expressing AIMP1/p43 and plasmid expressing C-E1-E2-V5 was transfected to Huh-7 cells. 24 hours' after transfection, cells were treated with 10 µM MG132 for 6 hours. Cells were immunostained with anti-AIMP1/p43 antibody (green) and anti-V5 antibody (red) and observed under confocal microscope. F. V5-tagged HCV E2 and AIMP1/p43 was observed in proximity ligation assay (PLA). Red dots show the interaction between E2 (either 2a or 1b type) and AIMP1/p43. Nucleus was stained with DAPI (blue).

HCV E2 is a viral membrane protein. As the virus acquires its membrane from endoplasmic reticulum, the protein is mainly found in endoplasmic reticulum and its vicinity. We tested whether E2 interacted with cellular AIMP1/p43 using GST pulldown and coimmunoprecipitation assay. GST-E2 fusion construct was made and expressed in Escherichia coli. The fusion protein was purified and used for GST pulldown assay. GST-E2 interacted with AIMP1/p43 while GST alone did not (Fig. 1A). The interaction inside the cell was confirmed by coimmunoprecipitation analysis. V5-tagged E2 was expressed in HEK 293 cell and coimmunoprecipitation was done using anti-AIMP1/p43 antibody (Fig. 1B). It was shown that E2 was coimmunoprecipitated with AIMP1/p43. Since E1 and E2 form a heterodimer after translation and proteolytic cleavage, we explored the interaction of E2 and AIMP1/p43 in the same situation. We expressed core-E1-E2 (C-E2-V5) from one plasmid and coimmunoprecipitation was done. When AIMP1/p43 was precipitated by anti-AIMP1/p43 antibody, V5 tagged E2 was detected while V5 tagged E1 (C-E1-V5) was not detected (Fig. 1C). Subcellular colocalization of the two proteins was observed from confocal microscopy analysis. The two proteins were mainly expressed in cytoplasm and closely localized when E2 was expressed alone (Fig. 1D) and entire structural proteins were expressed together (Fig. 1E). In addition, proximity ligation assay (PLA) showed the interaction of HCV E2 and AIMP1/p43 (Fig. 1F). In this assay, not only E2 form 2a type HCV but also E2 from 1b type HCV interacted with AIMP1/p43. Some E2s were seen apart from ER, possibly due to the overexpression. Thus, we could confirm that HCV E2 and AIMP1/p43 interacted each other.

### Endogenous AIMP1/p43 decreased by HCV E2

**Figure 2:**
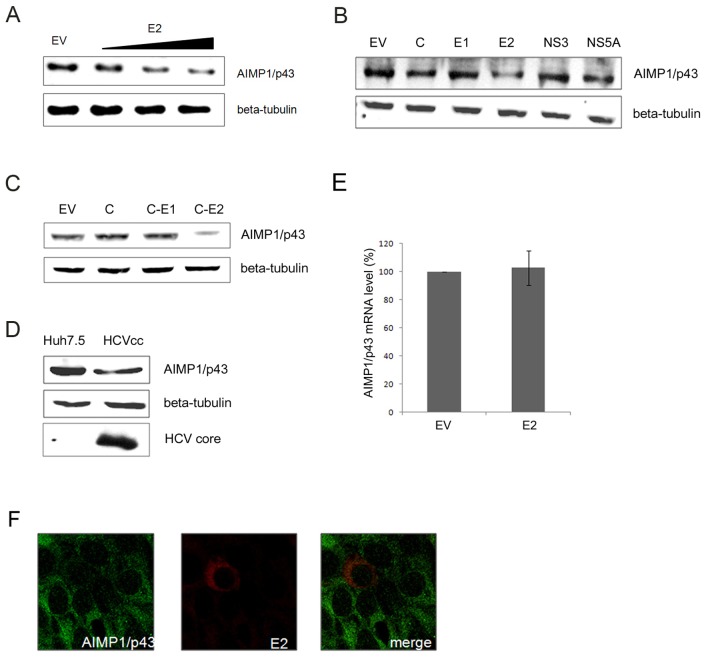
Endogenous AIMP1/p43 decreased in the presence of HCV E2. A. Plasmid expressing HCV E2 was transfected to Huh-7 cells and endogenous AIMP1/p43 was detected by anti-AIMP1/p43 antibody. The amount of plasmid used for transfection was 0.5, 1, and 2 µg for each lane. β-tubulin was used as a control. EV; empty vector. B. Plasmid expressing HCV core, HCV E1, HCV E2, HCV NS3, or HCV NS5A was transfected to Huh-7 cells and the change of amount of endogenous AIMP1/p43 was detected by anti-AIMP1/p43 antibody. C. Plasmid expressing HCV core, core-E1, or core-E1-E2 was transfected to Huh-7 cells and the enogenous AIMP1/p43 expression level was observed. D. JFH1 HCVcc was infected into Huh7.5 cells. The expression level of endogenous AIMP1/p43 was detected by anti-AIMP1/p43 and expression of HCV RNA was detected by anti-core antibody. E. Plasmid expressing HCV E2 was transfected to Huh-7 cells and expressed for 24 hours. Total RNA was isolated and real-time RT-PCR was performed. The average of three independent tests is shown with error bars. F. Plasmid expressing FLAG-tagged E2 was transfected to Huh-7 cells. 24 hours' after transfection, immunostaining was done using anti-FLAG antibody (red) and anti-AIMP1/p43 antibody (green). Cells were observed under fluorescencemicroscope.

When HCV E2 was expressed, endogenous AIMP1/p43 decreased in a dose-dependent manner (Fig. 2A). Other HCV proteins such as core, E1, NS3 and NS5A did not affect the level of AIMP1/p43 (Fig. 2B). When HCV core-E1-E2 were expressed from one plasmid, endogenous AIMP1/p43 decreased, while core-E1 did not affect AIMP1/p43 level (Fig. 2C). When HCV particle (HCVcc) was used to infect Huh7.5 cells, endogenous AIMP1/p43 decreased, too (Fig. 2D). To see whether the decrease was due to transcriptional control, AIMP1/p43 mRNA was quantified. Real-time reverse transcription PCR analysis revealed that the amount of AIMP1/p43 mRNA remained unchanged in the presence of HCV E2 (Fig. 2E). From a fluorescence microscopy analysis, we could observe that “endogenous” AIMP1/p43 level decreased in the cell expressing HCV E2 compared to adjacent cells that did not express E2 (Fig. 2F).

### HCV E2 induced ubiquitin-mediated proteasomal degradation of AIMP1/p43

**Figure 3:**
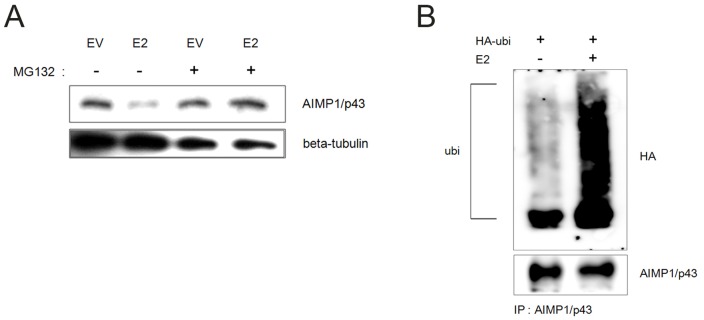
HCV E2 induced ubiquitin-mediated degradation of AIMP1/p43. A. Plasmid expressing HCV E2 was transfected to Huh-7 cells. Medium was changed 6 hours after transfection and MG132 was added at the concentration of 50 µM in 20 hours. Western blot analysis was done 6 hours after the treatment. β-tubulin was used as a loading control. EV; empty vector. B. Plasmid expressing HA-tagged ubiquitin and plasmid expressing HCV E2 were transfected to Huh-7 cells. Medium was changed 6 hours after transfection and MG132 was added at the concentration of 50 µM in 20 hours. Cell lysate was subjected to immunoprecipitation using anti-AIMP1/p43 antibody and protein G.

As AIMP1/p43 decreased in the presence of HCV E2, we tested if proteasomal degradation was involved. When MG132, a proteasome inhibitor, was added, the amount of AIMP1/p43 in the presence of HCV E2 increased suggesting that HCV E2 directed AIMP1/p43 to proteasomal degradation (Fig. 3A). Since most of proteasomal degradation was mediated by ubiquitination, we tested if HCV E2 induced ubiquitination of AIMP1/p43. In the presence of HCV E2, polyubiquitinated AIMP1/p43 was observed (Fig. 3B). Since HCV E2 did not possess an E3 ligase activity, it could recruit a protein that had E3 ligase activity to AIMP1/p43.

### Lowered expression of grp78 induced decrease of AIMP1/p43

**Figure 4:**
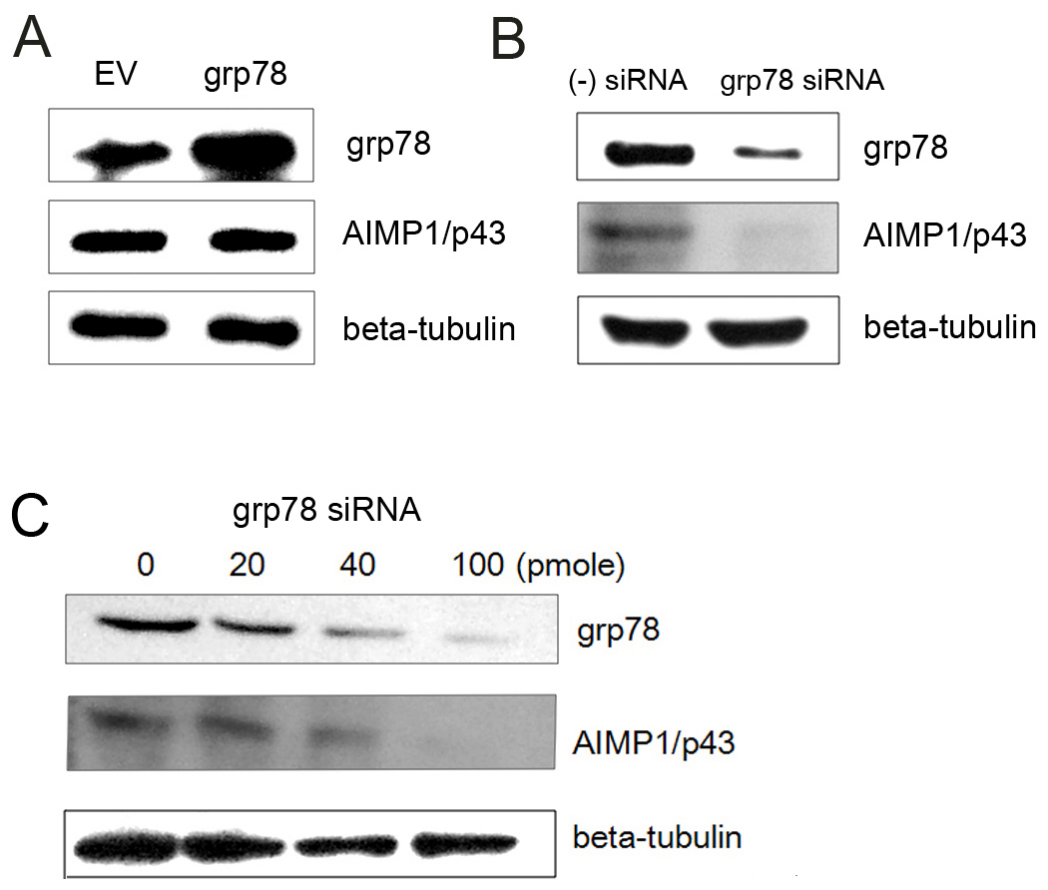
HCV E2 inhibited stabilization of AIMP1/p43 by grp78. A. Plasmid expressing grp78 was transfected to Huh-7 cells and grown. Cell lysate was subjected to western blot using anti AIMP1/p43 antibody and anti-grp78 antibody. β-tubulin was used as a loading control. EV; empty vector. B. grp78 siRNA or negative control siRNA was transfected to Huh-7 cells and grown. Cell lysate was subjected to western blot using anti AIMP1/p43 antibody and anti-grp78 antibody. C. grp7 siRNA was transfected to Huh-7 cells at indicated amounts. Cell lysate was subjected to western blot using anti AIMP1/p43 antibody and anti-grp78 antibody.

grp7, an ER chaperone, was reported to be overexpressed in the presence of HCV E2 [20]. Thus, we tested if grp78 exerted any effect on the level of AIMP1/p43. When grp78 was overexpressed in Huh-7 cells, no alteration of AIMP1/p43 level was observed (Fig. 4A). When grp78 siRNA was expressed for knockdown, AIMP1/p43 level decreased (Fig. 4B). The decrease of AIMP1/p43 was dependent on the amount of grp78 siRNA transfected (Fig. 4C). General decrease of translation resulting from unfolded protein response (UPR) in the presence of decreased grp78 could lead to decrease of general protein level including AIMP1/p43. But this possibility was normalized by showing a stable level of tubulin in Fig. 4B and C. Thus, the results suggest that grp78 stabilized AIMP1/p43 more than it did to other proteins.

### HCV E2 inhibited interaction between grp78 and AIMP1/p43

**Figure 5:**
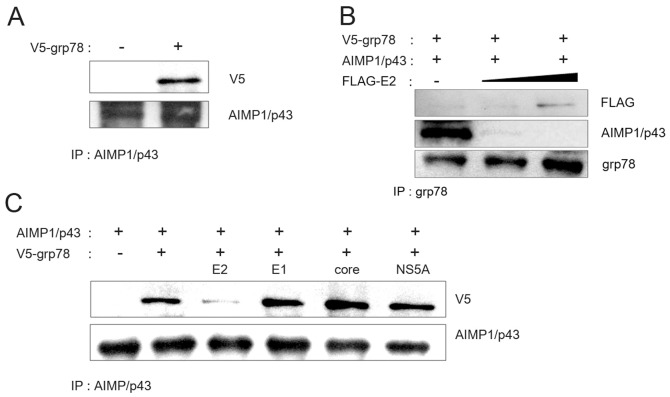
HCV E2 inhibited the interaction between grp78 and AIMP1/p43. A. Plasmid expressing V5-tagged grp78 and plasmid expressing AIMP1/p43 were transfected to HEK 293 cells and grown. Cell lysate was subjected to immunoprecipitation using anti-AIMP1/p43 antibody. B. Plasmid expressing V5-tagged grp78 and plasmid expressing AIMP1/p43 were transfected to HEK 293 cells along with 2 or 4 µg of plasmid expressing HCV E2 and grown. Cell lysate was subjected to immunoprecipitation using anti-AIMP1/p43 antibody. C. Plasmid expressing V5-tagged grp78 and plasmid expressing AIMP1/p43 were transfected to HEK 293 cells along with plasmid expressing HCV E2, E1, core, or NS5A and grown. Cell lysate was subjected to immunoprecipitation using anti-AIMP1/p43 antibody.

Above experiments showed that grp78 acted as a stabilizer for AIMP1/p43. Decrease of AIMP1/p43 could be either a simple additive effect of loss of stabilizing grp78 and expression of E2 inducing proteasomal degradation, or other interplay among the three proteins. It was already known that grp78 interacted with HCV E2 [20]. Thus, we tested if grp78 and AIMP1/p43 interacted each other. From a coimmunoprecipitation experiment we could observe that the two proteins precipitated together, suggesting an interaction in Huh-7 cells (Fig. 5A). In the presence of increasing amount of HCV E2, the interaction decreased in a dose-dependent manner (Fig. 5B). In the presence of HCV E2, the amount of coimmunoprecipitated grp78 with AIMP1/p43 notably decreased, while other HCV proteins including E1, core, and NS5A did not exert any effect (Fig. 5C). AIMP1/p43 should decrease in the presence of E2 by proteasomal degradation, thus AIMP1/p43 level was normalized in Fig. 5C. These results suggest that the stabilizing effect of grp78 to AIMP1/p43 was inhibited in the presence of HCV E2 by its blocking of the interaction between grp78 and AIMP1/p43.

### HCV E2 increased TGF-β signaling and cell surface expression of gp96

**Figure 6:**
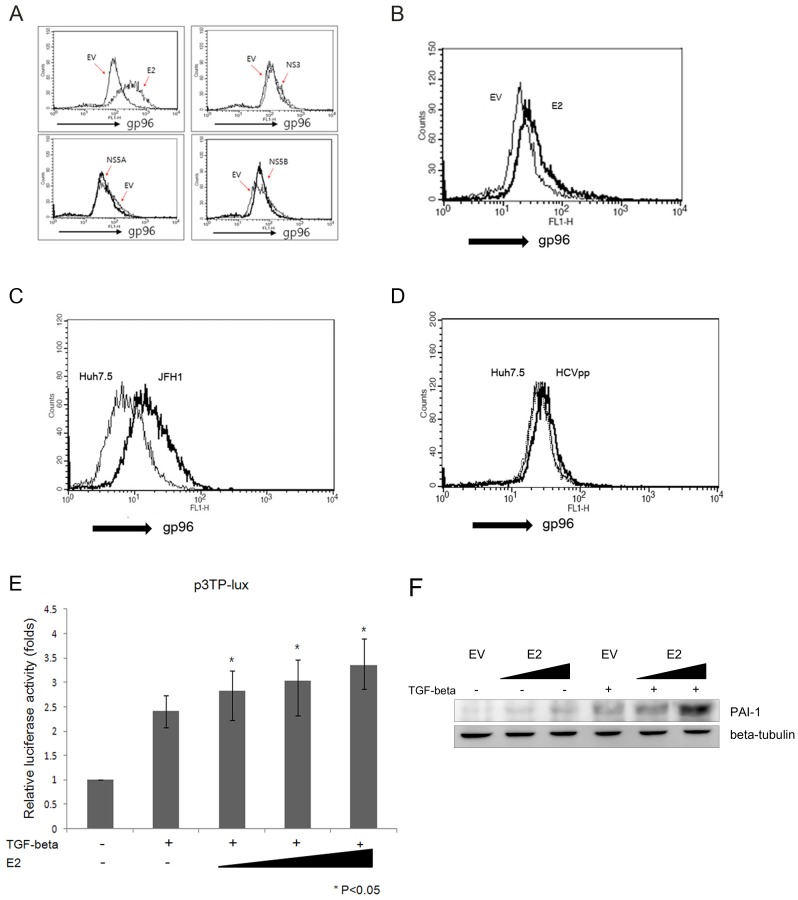
HCV E2 promotes cell surface expression of gp96 and TGF-β signaling. A. Palsmid expressing HCV E2, NS3, NS5A, or NS5B was transfected to Huh-7 cells and grown for 24 hours. Cell surface gp96 was probed with anti-gp96 antibody in flow cytometry. B. The same experiment with HCV E2 was carried out in Bjab cells. C. JFH1 particle (HCVcc) was infected to Huh7.5 cells. Cell surface gp96 was probed with anti-gp96 antibody using flow cytometry. D. HCV pseudoparticle (HCVpp) was infected to Huh7.5 cells. Cell surface gp96 was probed with anti-gp96 antibody using flow cytometry. E. A reporter plasmid, p3TP-lux, was transfected into Huh-7 cells along with 0.5, 1.0, or 2.0 µg of plasmid expressing HCV E2 and luciferase activity was measured in a luminometer. Transfection efficiency was normalized with cotransfection of pCH110 harboring LacZ gene and measuring β-galactosidase activity. Experiment was done in a triplicate. F. Plasmid expressing HCV E2 (either 0.5 µg or 2 µg) were used to transfect Huh-7 cells and PAI-1 expression was observed. TGF-β was added at the concentration of 4 ng/ml for 20 hours (lanes 4, 5, and 6) and PAI-1 expression was detected using western blotting with anti-PAI-1 antibody. EV; empty vector.

It is well known that HCV infection leads to autoimmune diseases and liver fibrosis. AIMP1/p43 was reported to stabilize smurf2 and act as a negative regulator of TGF-β signaling leading to liver fibrosis [19]. It was also reported that AIMP1/p43 inhibited Lupus-like autoimmune disease by decreasing cell surface expression of gp96 [12]. Since we showed that AIMP1/p43 was degraded in the presence of HCV E2 in two independent ways, we further explored whether HCV E2 had any effect on cell surface expression of gp96 and TGF-β signaling. When HCV E2 was expressed in Huh-7, surface expression of gp96 increased, while other HCV proteins such as NS3, NS5A, and NS5B had no effect (Fig. 6A). HCV was reported to infect lymphocytes in addition to hepatocytes [23]. When HCV E2 was expressed in Bjab, a B lymphocyte, we could also observe the increase of surface expression of gp96 (Fig. 6B). HCV particle produced from cell culture (HCVcc) also promoted cell surface expression of gp96 (Fig. 6C). However HCV pseudoparticle infection did not change the cell surface expression of gp96 (Fig. 6D). These data suggest that only intracellular E2 increased cell surface expression of gp96. In the presence of increasing amount of HCV E2, TGF-β signal increased in a dose-dependent manner (Fig. 6E). TGF-β induces expression of extracellular matrix proteins like collagen and PAI-1 [11]. HCV E2 upregulated TGF-β signaling and increased level of endogenous PAI-1 was observed (Fig 6F). These results suggest that HCV E2 could be a factor causing autoimmune diseases and liver fibrosis.

## Discussion

HCV infected individuals suffer from liver cirrhosis and autoimmune diseases in addition to chronic hepatitis [9], [32], [36]. It was recently revealed that AIMP1/p43 was involved in fibrosis and autoimmune diseases [19], [22]. Using the finding that HCV E2 interacted with AIMP1/p43, we presented evidences and possible mechanisms how HCV E2 caused liver fibrosis and autoimmune disease via interaction with AIMP1/p43 in this study.

Although HCV was reported to be related to autoimmune diseases, no clear explanation was given so far. gp96, a heat shock protein, plays roles as a molecular chaperone and as a mediator of immune responses [37]. gp96 interacts with CD91 and TLR2/4 of dendritic cells and induces cell maturation [39]. Cell surface expression of gp96 is reported to induce activation of dendritic cells and spontaneous autoimmune diseases [22]. AIMP1/p43 was reported to retain gp96 in the ER so that the surface expression of gp96 decreased [12]. We confirmed E2 expression caused decrease of AIMP1/p43 expression level which increased cell surface gp96. From the data presented in this study, we suggest a possibility of induction of autoimmune disease by HCV E2.

HCV causes chronic hepatitis and further leads to liver cirrhosis and hepatocellular carcinoma [3], [13], [14]. HCV core and NS5A induce alteration of lipid metabolism. Then they induce steatosis and production of ROS. ROS leads to induction of TGF-β, leading to liver fibrosis. The major cytokine involved in fibrosis is TGF-β and its signaling induces production of extracellular matrix (ECM) proteins [11]. In this study, E2 was also shown to increase TGF-β signaling. E2 expression lead to degradation of AIMP1/p43 regulating TGF-β signaling negatively and, as a result, E2 expression increased TGF-β signaling. Similarly, HDV LHDAg (large hepatitis delta antigen) and HBV pX protein induce liver fibrosis by increasing TGF-β signaling [6], [16]. Also, TGF-β signaling has a cancer suppressive activity through inducing growth arrest and apoptosis [31]. HCV core, NS3, and NS5A are reported to inhibit TGF-β signaling and to induce hepatocarcinogenesis [5], [7]. Others report they activate JNK (c-Jun N-terminal kinase) which induces fibrogenesis with TGF-β signaling [24], [40]. At present, it is not clear how the viral proteins regulate TGF-β signaling differently. It could be stage-specific during the progression of the disease.

Since HCV acquires its membrane from endoplasmic reticulum, produced E2 protein is targeted to ER and retained in pre-Golgi compartment without being secreted [21]. Thus, E2 induces ER stress and ER chaperones are produced [20]. grp7 acts as an UPR (unfolded protein response) protein. When ER stress is induced in the presence of unfolded or misfolded proteins in ER, UPR proteins are produced [1]. UPR sensors are membrane proteins such as IRE1, PERK, and AFT6 [34]. They are bound to grp78 and inactive in the absence of ER stress. As the amount of unfolded proteins increases, grp78 is detached and sensors transduce UPR signal to each pathway. It leads to lowered translation and increased ER-associated protein degradation. Eventually, protein folding ability and secretion potential is increased [1], [34]. We showed that grp78 interacted with and stabilized AIMP1/p43. HCV E2 was reported to interact with grp78 [20]. Since we showed interaction between E2 and AIMP1/p43, all three proteins interacted with each other. From the data presented in this study, we suggest a mechanism where E2 and grp78 competes for binding to AIMP1/p43. In the presence of E2, grp78 binding to AIMP1/p43 decreases thus losing its stabilizing effect. As a summary, HCV E2 causes the degradation of AIMP1/p43. E2 inhibits the interaction between grp78 and AIMP1/p43 in which grp78 stabilized AIMP1/p43 and leads to proteasomal degradation via ubiquitination. This mechanisms lead to a decrease in the cellular level of AIMP1/p43, causing increased TGF-β signaling and increased cell surface expression of gp96.
